# The Roll of Crime

**Published:** 1861-02

**Authors:** 


THE following from the editorial of the *Am. Med. Times* contains thoughts worthy of the attention of all classes and professions.—[*Ed. Register.*

THE ROLL OF CRIME.—During the year 1860, just closed, 116 citizens of the city of New York died by the hand of violence. Of this number, 59 are recorded as homicides, and 57 as suicides.

The problem of the prevention of crime has taxed the genius of the wisest statesmen and the most experienced philanthropists. To this end the penitentiary, the prison, the rack, and the gallows have been established, but as yet without avail in completely restraining the vicious. With reference to homicide, this question presents two phases: 1st, The removal of the cause of crime; 2d, The punishment of the criminal. It will surprise no one to learn that on investigation it appears that in the great majority of cases of homicide, intemperance is the cause. In this city, so distinguished for its "rum for the million," it supplies the animus to the criminal, however thoroughly his plans are premeditated, in nine cases out of ten. This fact is so patent to every observer that it needs no illustration at our hands. But one plain, simple, practical question presents itself to the legislator, viz. shall this prolific cause of the most heinous crime known to human society, be removed? On the answer to this question depends the length of our criminal calendar. We are aware that many difficulties tend to complicate its settlement in the affirmative, but we are also aware that these obstacles have been met by other communities, and resolutely overcome. The results of such legislation have always been of the most cheering character. Penitentiaries, prisons, and almshouses, have been deprived of their occupants, and even courts have met to adjourn without a cause on their criminal calendar. No man can doubt that if during the year upon which we have entered, not a drop of spirituous liquor was drunk by the people of this city, our almshouse, hospitals, and prisons would be emptied of nine-tenths of their present number of inmates, and our criminal statistics for the year would be reduced 99 per cent.

In the correction of criminals, the first impulse of government was to appeal to the fear of men, and hence have been instituted the most frightful punishments. While the more simple offenses growing out of avarice and kindred propensities were thus checked, the more heinous crimes, which are the result of violent and intensely stimulated passion, received but little restraint. Subsequently a more philosophical study of criminal jurisprudence discovered the fact that vicious men are restrained rather by the certainty, than the severity of punishment. This led to important discriminations in the degrees of crime, and corresponding modifications in the severity of the penalties. This principle should never be lost sight of in legislation for the suppression of crime.

But with the progress of human knowledge and practical Christian philanthropy, new opinions have been formed of man's moral nature, and of his relations to his Creator and his fellow men, which are yet to lead to the most important modifications of our criminal laws. The question, should not all punishments be so modified as to be reformatory of the individual? is already receiving a practical solution in many States. The final prevalence of the conviction, that the period of restraint of the criminal should be taken advantage of by the State for his reformation, that he may be returned to society a good citizen, will be the grandest triumph of a Christian civilization.

The prevention of suicide involves also two points, viz. 1st, The removal of its causes; 2d, The removal of the means by which it is accomplished. The alleged causes of suicide are numerous. They are insanity, intemperance, melancholy, disappointment, revenge, &c. If, however, each case were carefully investigated, we doubt not these causes with due discrimination might, for the most part, be reduced to one, viz: insanity. The researches in psychological medicine have established the fact that insanity lurks in the community in concealed forms; while all are cognizant of its sudden development in the perpetration of shocking crimes. There can be no doubt that many who are actively engaged in business, or walk the streets, or mingle in society, have those mental proclivities which the most trifling perturbating causes would so unbalance as to lead to personal violence. Most physicians can recall instances of the self-destruction of persons, who, on reflection, they recollect have exhibited many singular peculiarities to which they did not attach sufficient importance. Toward this class of suicides our profession has a most important duty to perform. They should be more thorough in the investigation of the secret springs of melancholy, disappointment, or other disturbing influences of the mind and passions, and so far as possible remove them. Had this been done in numerous cases recorded in this list, it is evident many lives would have been saved, and much human suffering and misery prevented.

Of the means by which suicide is perpetrated, there is but one class over which we have control, viz: poisons. The law of this State is now sufficiently stringent to prevent the sale of poisons to irresponsible persons, *provided* it is enforced. But it is a melancholy fact that of these fifty-seven suicides, twenty-four accomplished self-destruction by poisons. On the druggists of New York falls the fearful verdict of TWENTY FOUR MURDERS IN THE YEAR 1860. What answer have they to make why sentence should not be pronounced?

